# Enhanced Hyaluronic Acid Production in *Streptococcus equi* subsp. *Streptococcus* *zooepidemicus* Through Strain Mutagenesis and Optimized Purification

**DOI:** 10.5812/ijpr-164108

**Published:** 2025-09-13

**Authors:** Kamand Hedayat, Delaram Doroud, Naser Mohamadpour Dounighi, Mohammad Ali Shokrgozar, Maryam Shahali, Mohammad Hassan Houshdar Tehrani

**Affiliations:** 1 Department of Biotechnology, Production and Research Complex, Pasteur Institute of Iran, Tehran, Iran; 2 Department of Immunotherapy and Leishmania Vaccine Research, Pasteur Institute of Iran, Tehran, Iran; 3 Department of Human Vaccines and Serum, Razi Vaccine and Sera Research Institute, Karaj, Iran; 4 Department of Cell Bank, Pasteur Institute of Iran, Tehran, Iran; 5 Research and Production Complex, Pasteur Institute of Iran, Tehran, Iran; 6 Department of Medicinal Chemistry, School of Pharmacy, Shahid Beheshti University of Medical Sciences, Tehran, Iran

**Keywords:** Hyaluronic Acid, Purification, Random Mutagenesis, Strain Optimization, *Streptococcusequi* subsp. *S. zooepidemicus*

## Abstract

**Background:**

* Streptococcusequi* subsp. *S. zooepidemicus* is a notable producer of hyaluronic acid (HA), a natural polysaccharide widely utilized in the pharmaceutical, cosmetic, and food industries due to its unique properties.

**Objectives:**

This study focuses on strain optimization and the purification of HA. The key goals include reducing hyaluronidase activity, which degrades HA, eliminating beta-hemolytic traits, and developing a purification process that significantly minimizes organic solvent consumption to lower costs and reduce environmental impact.

**Methods:**

UV-induced physical mutagenesis and chemical mutagenesis utilizing nitrous acid and N-methyl-N'-nitro-N-nitrosoguanidine (NTG) were employed to generate mutant strains with diminished hyaluronidase activity and beta-hemolytic properties. Following strain optimization, an efficient downstream process was implemented, focusing on pre-treatment methods involving pH and temperature adjustments, followed by ultrafiltration and phenol acetate treatment.

**Results:**

The selected mutant, designated as *S.* *equi* subsp. *S. zooepidemicus* K12, maintained stable mutation characteristics over 15 generations. The HA yield of the selected mutant showed an increase of 85.7%, rising from 0.42 g/L in the wild type to 0.78 g/L. According to gel permeation chromatography, the average molecular weight (Mw) of HA increased from 6.7 × 10^4^ Da to 1.2 × 10^5^ Da. Our purification strategy achieved a recovery rate of 72% with approximately 0.3% protein impurities, meeting European Pharmacopoeia (EP) standards. Additionally, organic solvent consumption was reduced by at least 25-fold compared to conventional methods.

**Conclusions:**

The study presents an integrated approach to HA production, encompassing strain improvement and purification. It addresses key challenges in the manufacturing of streptococcal HA and contributes to the development of more efficient and environmentally friendly purification methods, ultimately resulting in high-purity HA.

## 1. Background

Hyaluronic acid (HA) is a naturally occurring linear anionic polysaccharide composed of 2,000 to 25,000 disaccharide repeating units of D-glucuronic acid and N-acetylglucosamine, with a Mw range of 10^3^ Da to 10^7^ Da. The HA acid can absorb water many times its own weight. This property makes it an effective lubricant in the pharmaceutical, cosmetic, and food industries. Various biomedical applications of this biodegradable, non-immunogenic, and biocompatible polymer include the treatment of osteoarthritis, ophthalmic surgery, wrinkle-preventing fillers, wound healing, and tissue engineering (1, 2).

Previously, HA was primarily extracted from rooster comb, a process that posed significant challenges due to a high risk of contamination. Consequently, bacterial sources, particularly *streptococcus* groups A and C, have become the main producers of this polymer (3, 4). *Streptococcus* *equi* subsp. *S. zooepidemicus* is a notable producer of HA; however, it faces two key challenges. First, these bacteria produce hyaluronidase, an enzyme that degrades HA, which is undesirable for HA production. Second, *S. zooepidemicus* exhibits beta-hemolytic properties, meaning it can lyse red blood cells (5). Random mutagenesis is commonly applied to overcome these two undesirable traits. Three commonly used mutagens are UV radiation, nitrous acid, and N-methyl-N'-nitro-N-nitrosoguanidine (NTG), chosen for their different mutation mechanisms to increase the chance of obtaining desirable mutants. UV radiation is a physical mutagen that induces DNA damage by forming pyrimidine dimers, causing point mutations, deletions, and substitutions. Nitrous acid acts chemically by deaminating DNA bases, converting cytosine to uracil and adenine to hypoxanthine, leading to point mutations. The NTG alkylates DNA bases, resulting in various mutations including point mutations and frameshifts. These mutagenesis methods have been successfully applied to *S. zooepidemicus* (6) and *Streptococcus* *equisimilis* (7) to produce mutants with increased HA yield and Mw, as well as reduced hyaluronidase activity and beta-hemolytic properties, which are important for improving HA production quality and quantity.

After obtaining the desired strain, designing the optimal downstream method to achieve high-purity HA for medical applications with the desired Mw is considered one of the most important steps in the bioengineering of this product. Proteins are the main impurities in microbial HA production. To date, many HA purification approaches have been used, and nearly all strategies are primarily based on organic solvent precipitation. Precipitation as the initial stage of purification in many studies is often carried out by frequent organic solvent precipitation or by the use of quaternary salts (8-10). The purification process often involves a combination of techniques such as ultrafiltration, adsorption using activated charcoal, and precipitation with organic solvents like ethanol or isopropanol (11, 12). Despite the ease and efficiency of solvent precipitation, the need for a high volume of organic solvents and consecutive precipitation steps to achieve high purity has increased the desire to replace or at least reduce this method with cheaper and environmentally friendly alternatives. The use of activated charcoal can be limited by its potential to introduce residual contaminants if not properly cleaned, and it may not effectively remove all impurities, particularly proteins (13). Additionally, silica gel, another common adsorbent, can be expensive and may require multiple filtration steps, increasing the complexity and cost of the purification process (14). Given these challenges and the increasing market demand for HA, there is an urgent need to develop more efficient purification methods for industrial applications. In general, with an increase in the number of purification steps to reach the desired purity, in addition to the complexity of the downstream process, the structure of the polymer is exposed to change, and the HA recovery rate will decrease (15).

For HA production in *S. zooepidemicus*, to overcome the two key challenges of hyaluronidase activity and beta-hemolytic properties, this study employed random mutagenesis techniques. Specifically, UV-induced physical and chemical mutagenesis with nitrous acid and NTG was utilized to generate strains of *S. zooepidemicus* with diminished hyaluronidase activity and beta-hemolytic properties, thereby enhancing the yield and increasing the Mw of the produced HA. To evaluate the effects of mutagenesis, the coding region of hyaluronan synthase, spanning upstream of hyaluronan synthase A (hasA) to hasC, was sequenced and compared between the wild-type and mutant strains.

## 2. Objectives

Our study proposes a novel downstream process focusing on pH and temperature adjustments followed by ultrafiltration and phenol acetate precipitation. This approach minimizes organic solvent consumption while maintaining the high purity and structural integrity of HA. By optimizing these conditions, we can reduce the complexity of the purification process and enhance the recovery rate of HA, ultimately producing high-purity grade HA with the desired Mw. The advantages of our method include reduced environmental impact, lower operational costs, and the potential for higher yields and purity compared to traditional methods.

## 3. Methods

### 3.1. Microorganism and Culture Media

In this study, a wild-type *S. equi* subsp. *S. zooepidemicus* strain isolated from the nasal swab of a horse in Mashhad, Iran (16), served as the primary subject of our investigation, which we registered in the NCBI database under the designation K1. The microorganism was cultivated in brain heart infusion (BHI) medium. To identify non-hemolytic mutants, blood agar medium was employed as a detection method. The BHI medium supplemented with 0.1% w/v HA was used to screen for hyaluronidase-negative mutants. For the selected mutant, the chosen fermentation medium consisted of 70 g sucrose L^-1^, 25 g yeast extract L^-1^ (Merck, cat number 103753), 1.3 g K_2_SO_4_ L^-1^, 2 g MgSO_4_·7H_2_O L^-1^, 6.2 g Na_2_HPO_4_·12H_2_O L^-1^, and 0.005 g FeSO_4_·7H_2_O L^-1^. For small-scale seed cultures, 10 mL of the medium is inoculated and incubated at 37°C with 220 RPM agitation. Intermediate scaling involves transferring 10% (5 mL) of this seed culture into 50 mL of production medium in a 200 mL Erlenmeyer flask, maintaining similar conditions. Finally, for large-scale fermentation, 10% (30 mL) of the intermediate culture is transferred into 5 separate 3 L flasks containing 0.6 L of the defined production medium. The fermentation runs for 24 hours under controlled conditions, including periodic pH adjustments using sterile NaOH or HCl to maintain the target range (7 - 7.2), ensuring optimal HA synthesis. Aeration is facilitated using breathable closures such as cotton plugs, promoting passive oxygen transfer throughout the process.

### 3.2. Bacterial Suspension Preparation

To prepare the bacterial suspension, a single clone was cultured overnight in 5 mL of BHI medium, subsequently transferred to 50 mL of fresh BHI medium, and incubated for 6 hours, corresponding to the mid-logarithmic phase of the growth curve. The bacterial pellet was collected by centrifugation at 8,000 g for 20 minutes at 4°C, washed twice with sterile physiological saline, and re-suspended in the same solution to match the McFarland 0.5 standard. Before mutagenesis, a control sample was prepared and incubated at 37°C. To determine the exposure dose that results in 99.99% lethality, the reduction in the number of colonies grown on a BHI agar plate under exposure was compared to the control plate. A 99.99% reduction corresponds to a 4-log reduction in colony count.

#### 3.2.1. UV Mutagenesis

The prepared bacterial suspension was spread in a thin layer on sterile plates. These plates were then placed in the dark, 20 cm away from a 20-watt UV lamp emitting at 254 nm. The samples were irradiated for varying durations of 30, 60, 90, 120, and 150 minutes. Following irradiation, the bacterial suspensions were diluted to the same extent as the control sample, and plates were incubated in the dark until colonies appeared. Finally, the number of colonies on the irradiated plates was compared to the control plate. The UV dose resulting in 99.99% lethality was selected as the optimal dose for subsequent experiments.

#### 3.2.2. Chemical Mutagenesis

For nitrous acid mutagenesis, a bacterial suspension was prepared in 0.2 M acetate buffer (pH 4.5) to match the McFarland 0.5 standard, similar to the UV mutagenesis method. The bacterial suspension was subjected to mutagenesis using nitrous acid at concentrations of 0.2, 1, and 2 M for durations of 10, 30, and 60 minutes. This process was conducted with shaking at 150 RPM and 37°C. To terminate the reactions, phosphate buffer (0.2 M, pH 7.2) was added at a 4:1 ratio. For NTG mutagenesis, the bacterial suspension was prepared in phosphate buffer (2 M, pH 7) using the 0.5 McFarland method and exposed to an NTG concentration of 400 µg/mL for varying times of 10, 25, 40, and 60 minutes. To terminate the reaction, a 0.16 M sodium thiosulfate solution was added at a 10:1 ratio. The exposure time and concentration resulting in 99.99% lethality were selected as the optimal dose for subsequent experiments.

### 3.3. Mutant Selection

Mutant selection was conducted to choose from a diverse pool of mutants. Based on the study by Kim et al. (17), among the identified strains exhibiting both desired phenotypes, clones exhibiting bigger viscous capsules were prioritized, as larger capsules are associated with a higher probability of increased HA production. The final selection of the mutant was based on the carbazole assay (18), which measured HA production, ensuring that the chosen strain not only displayed the required phenotypic traits but also had enhanced production capabilities. The genetic stability was evaluated over 15 generations, as a compromise between previous studies assessing 25 (6) and 10 generations (7), to strike a balance in the evaluation timeframe.

### 3.4. DNA Purification, Amplification, and Has Operon Sequencing of Selected Mutant and Wild Type

To evaluate the effects of mutagenesis in DNA purification for hyaluronan synthase gene sequencing, genomic DNA was extracted from the samples by boiling. The concentration and purity of the extracted DNA were assessed using a NanoDrop^™^ 2000c spectrophotometer (Thermo Fisher Scientific). For amplification, four pairs of primers were designed for the target region as mentioned in Table 1. The PCR reaction mixture included 10 µL of 2X PCR Master Mix, DNA PFU polymerase (Kiagene Fanavar Aria Co.), 0.7 µL of each forward and reverse primer, and 5 µL of template DNA, topped up with nuclease-free water to a final volume of 20 µL. The thermal cycling conditions were set to an initial denaturation at 93°C for 3 minutes, followed by 35 cycles of denaturation at 93°C for 30 seconds, annealing at the optimal temperature determined for the primers, which was 60°C for 45 seconds, and extension at 72°C for 80 seconds. A final extension step was performed at 72°C for 10 minutes. The PCR was conducted using a Biometra T-Gradient thermal cycler (Analytik Jena). The amplified products were analyzed via agarose gel electrophoresis to confirm successful amplification before proceeding with sequencing.

**Table 1.** Primers for Amplification of the Has Operon

No.	F (5’→3’)	R (5’→3’)
**1**	GACTTGGTGAGCATAGGCAAATC	GCATAGTTGGTCAAGCACCTGTC
**2**	TAATATCCTTGTTTGCTCAGGTC	TCAATATCAGCATCCTTGATTGG
**3**	GCTCTATAAAGACGAACCAGAGG	CAACCCTGTAGCCAATATCCTCG
**4**	ATAGAGGCATTCCCCAGTCCTTG	AACTTGTCCCCAACATCATACCG

### 3.5. Separation and Purification of Hyaluronic Acid

#### 3.5.1. Pre-treatment and Bacterial Separation

To investigate the effect of pre-treatment methods on protein impurity, after controlled cultivation from the selected mutant, the culture was subjected to one of the following pre-treatment protocols:

1. SDS Pre-treatment: The culture was treated with SDS at concentrations of 0.2%, 0.1%, or 0.05% w/v for 10 minutes.

2. Tween 20 pre-treatment: The culture was treated with Tween 20 at varying concentrations of 0.1%, 0.2%, and 0.4% w/v for 10 minutes.

3. Triton X100 pre-treatment: The culture was treated with Triton X100 at varying concentrations of 0.1%, 0.2%, and 0.4% w/v for 10 minutes.

The efficacy of these pre-treatment methods was evaluated based on their ability to remove proteins in comparison with no pre-treatment. Protein concentration was assessed using the Bradford test. Subsequently, the broth was subjected to heat at 70°C for 1 hour, followed by cooling to room temperature. Following this step, the biological solids were separated from the broth using centrifugation at 10,000 g for 15 minutes.

#### 3.5.2. Purification

##### 3.5.2.1. pH and Temperature Adjustment

Following the separation of the biological solids, the culture underwent a first purification step involving pH adjustment.

1. TCA pre-treatment: The culture was treated with TCA at concentrations of 5%, 2.5%, or 1% w/v for 2 hours.

2. Citrate buffer pre-treatment: The culture was treated with citrate buffer at varying concentrations of 0.1%, 0.2%, and 0.4% w/v for one hour.

After adjusting the pH for protein aggregation, to investigate the effect of temperature, it was maintained at either 4°C or 60°C for one hour, and protein removal was evaluated using the Bradford test. Finally, to separate proteins and aggregated impurities, the solution was centrifuged at 10,000 g for 15 minutes, and the supernatant was collected for the next step.

##### 3.5.2.2. Diafiltration

For the removal of remaining soluble impurities related to the culture medium and proteins, an ultrafiltration process was employed using a Millipore TFF device with 50 kDa cut-off filters (polyether sulfone) in diafiltration mode. Distilled water was added as the diafiltrate, maintaining equal exit flux, and filtration continued until reaching a volume concentration ratio (VCR) of 4. The ultrafiltration was conducted at room temperature, adopting process conditions based on Zhou's proposed method: Transmembrane pressure of 0.15 MPa, solution pH of 7, and ionic strength of 0.25 M NaCl (19). Samples were collected from the retentate after each diavolume exchange to examine protein removal; finally, the retentate containing HA was collected for the next step. Post-ultrafiltration, the system was cleaned with 0.2 M NaOH and rinsed with distilled water until the permeate pH reached 7.

##### 3.5.2.3. Phenol Acetate Treatment

Following the ultrafiltration stage, the final volume was reduced to a quarter of the initial volume for the final polishing step of purification; a phenol acetate treatment was employed. The ultrafiltration-achieved solution containing HA was mixed with an equal volume of phenol acetate buffer. This mixture was subjected to gentle shaking for 30 minutes at 4°C. The phenol acetate buffer was prepared by combining a 3% w/v sodium acetate solution (10% w/v) with phenol, and the final pH was adjusted to 7.00.

### 3.6. Analytical Methods and Calculations

#### 3.6.1. Bitter and Muir Method for Hyaluronic Acid Concentration Measurement

The concentration of HA is determined using the Bitter and Muir method (18), which is based on the reaction of glucuronic acid with carbazole reagent. To prepare the sample for this assay, the fermentation broth is first centrifuged to remove cells. Subsequently, HA is precipitated from the supernatant by adding two volumes of ethanol. The precipitated HA is washed three times with ethanol to remove glucose and other soluble sugars that could react with carbazole and interfere with the assay by producing unwanted colors. After the washing steps, the precipitate is re-dissolved in 0.15 M sodium chloride, yielding purified HA for analysis. In the Bitter and Muir method, HA undergoes hydrolysis in the presence of acid, resulting in the cleavage of the glucuronic acid ring. This reaction produces a colored complex upon the addition of carbazole, which can be quantified spectrophotometrically at 530 nm. The concentration of HA was calculated using a standard curve generated with D-glucuronic acid, and the HA yield was then determined by multiplying this concentration by the sample volume using the equation Yield = C × V.

#### 3.6.2. Macro Bradford Method for Protein Concentration Determination

The protein concentration during each purification step was assessed using the Bradford method (20), a widely adopted colorimetric assay known for its simplicity and sensitivity. This method relies on the binding of Coomassie Brilliant Blue G-250 dye to proteins, resulting in a shift in the dye's absorbance maximum from 465 nm to 595 nm as it transitions from a red to a blue form upon binding. The procedure involves mixing the protein sample with Bradford reagent, measuring the absorbance at 595 nm after a brief incubation period, and comparing the absorbance to a standard curve created from known concentrations of bovine serum albumin (BSA). To calculate the protein reduction percentage, the following formula was used: Percentage protein reduction = (CPi / CP initial) × 100 (CPi: Protein concentration at each stage, CP initial: Initial protein concentration at the start).

#### 3.6.3. Characterization of Purified Hyaluronic Acid Using Fourier Transform Infrared

To characterize purified HA, Fourier transform infrared (FTIR) spectroscopy (Bruker Tensor 27) was employed within a scan range of 400 to 3000 cm^-1^.

#### 3.6.4. Molecular Weight Determination via Size Exclusion Chromatography

The Mw of HA is determined using size exclusion chromatography (Agilent 1100) coupled with a Refractive Index detector. In this process, purified HA samples were separated based on their size using aquagel-OH 8 µm mixed-H 300 × 7.5 mm columns with pullulan standards. The mobile phase consisted of NaNO_3_ 0.2 M + NaH_2_PO_4_ 0.01 M at pH = 7, flowing at a rate of 1 mL/min while maintaining a column temperature of 35°C. By comparing retention times against known standards, one can accurately estimate the Mw distribution of HA, which is critical for assessing its functional properties in biological applications.

#### 3.6.5. Protein Impurity Determination in Final Purified Hyaluronic Acid According to European Pharmacopoeia Methods

For the determination of protein impurities in purified HA powder, methods outlined by the European Pharmacopoeia (EP) (21) are used. This involves measuring protein concentrations through a reaction with cupri-tartaric acid, in which proteins form a complex that can be quantified spectrophotometrically at 750 nm. The absorbance readings were compared with those obtained from BSA standards, allowing for precise determination of protein levels in HA samples.

#### 3.6.6. Gel Permeation Chromatography for Protein Impurity Profile

To further evaluate protein impurities relative to HA peaks, gel permeation chromatography was performed using a hydrogel 2000 column (300 × 7.8 mm) as the stationary phase and 0.1 M sodium nitrate as the mobile phase at a flow rate of 0.8 mL/min at 70°C. A sample volume of 20 µL was injected into this analytical setup equipped with UV and Refractive Index detectors for simultaneous detection of proteins and HA, respectively. This dual-detection approach allows for comprehensive profiling of both components, thereby facilitating an understanding of their interactions and purity within the sample matrix.

#### 3.6.7. Measurement of DNA Impurities

To assess DNA contamination levels, 100 mg of freeze-dried HA was dissolved in a 9 g/L sodium chloride solution. The sample underwent 12 hours of low-intensity orbital mixing to ensure homogeneity. UV-Vis spectrophotometric analysis was then performed at 260 nm to quantify the nucleic acid content.

#### 3.6.8. Measurement of Phenol Residues

In accordance with the EP, residual phenol was quantified using standard solutions containing 5 to 30 µg/mL phenol. The test and standards were reacted with 4-aminoantipyrine and potassium ferricyanide, and after 10 minutes, the absorbance was measured at 546 nm to calculate phenol content (21, 22).

#### 3.6.9. Moisture Content Measurement via Loss-on-Drying

The moisture content of purified freeze-dried HA was assessed using the Loss-on-Drying (LOD) test. In this procedure, approximately 0.5 grams of purified HA are dried at temperatures ranging from 100°C to 110°C until a constant weight is achieved. The difference in weight before and after drying provides an accurate measure of moisture content.

## 4. Results

### 4.1. Mutation

**Figure 1.**
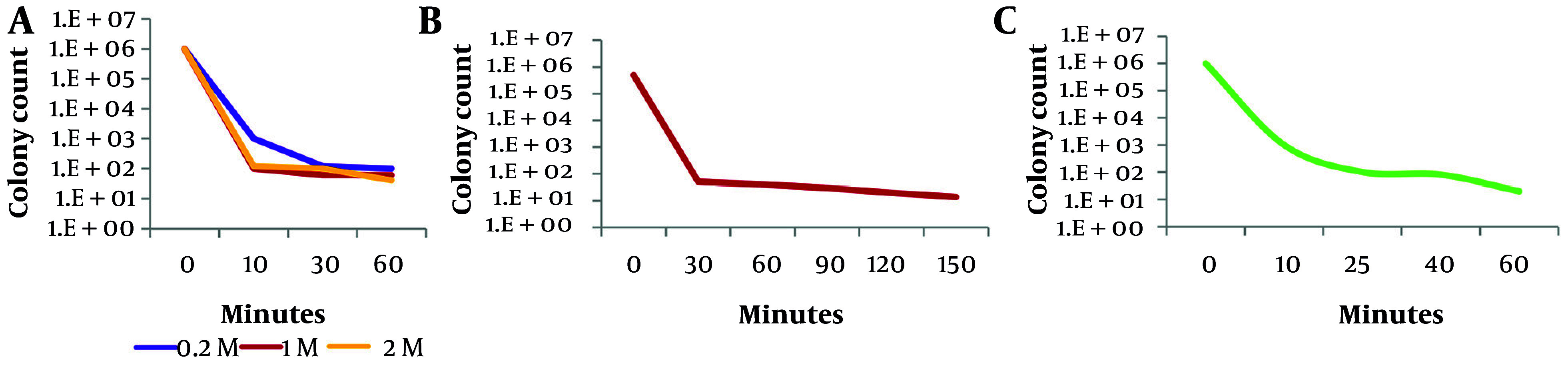
Lethality dose determination following exposure to (A) nitrous acid, (B) UV, and (C) N-methyl-N'-nitro-N-nitrosoguanidine (NTG)

To determine a lethal dose corresponding to 99.99% lethality, the samples were exposed to three mutagens: UV radiation, nitrous acid, and NTG. As illustrated in Figure 1, treatment with 1 M nitrous acid for 10 minutes (A), exposure to UV light for 30 minutes (B), and exposure to 400 µg/mL NTG for 25 minutes (C) resulted in 99.99% lethality.

### 4.2. Mutant Selection

Out of the seven mutants exhibiting the desired phenotypic characteristics, three were derived from the NTG mutation, while the others resulted from a combination of the NTG and UV mutations. One mutant (N-3-26) exhibited no increase in HA yield, while two others exhibited a decrease in production levels (N-2-17, NU-2-7). The four mutants with significant increases in HA production showed enhancements of 12.3%, 17.8%, 21.1%, and 85.7%, respectively (Table 2). The mutant exhibiting the highest increase of 85.7% in HA production was selected for further analysis, confirming its superior performance and stability regarding beta-hemolytic and hyaluronidase-negative traits throughout 15 sub cultivations.

**Table 2.** Percentage Changes in Hyaluronic Acid Production in Mutant Strains Compared with the Wild-Type Strain ^a^

**Mutant Name**	HA Titer (mg/mL)	HA % Titer Change to Wild Strain
**Wild strain**	0.42 ± 7.14	-
**N-2-17**	0.34 ± 5.88	17.6 ⬇
**N-3-26**	0.43 ± 9.41	0.03 ≈ 0
**N-3-57**	0.49 ± 8.59	17.8 ⬆
**NU-1-36**	0.78 ± 7.16	85.7 ⬆
**NU-1-57**	0.50 ± 10.88	21.1 ⬆
**NU-2-7**	0.22 ± 3.40	47.1 ⬇
**NU-2-101**	0.47 ± 1.34	12.3 ⬆

Abbreviation: HA, hyaluronic acid.

^a^ Values are expressed as mean ± SD.

### 4.3. Sequencing Results

**Figure 2.**
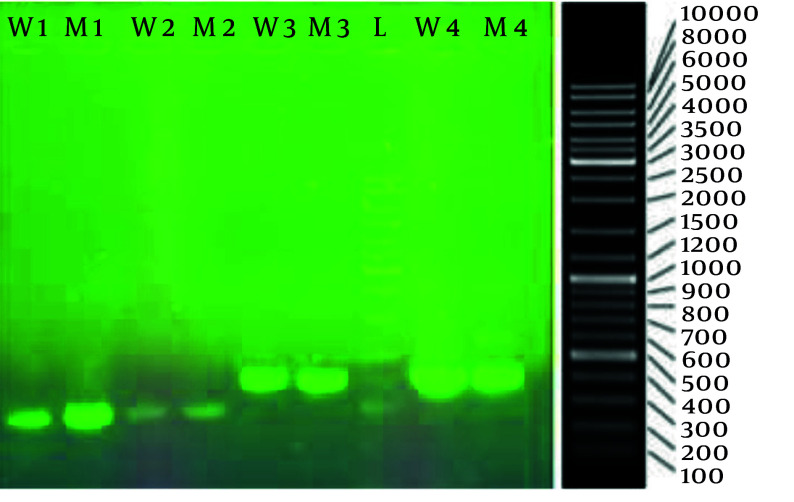
Agarose gel electrophoresis result of the hyaluronan synthase gene PCR product, from left to right; lane 1 and 2: Primer 1 for wild and mutant K12; lane 3 and 4: Primer 2 for wild and mutant K12; lane 5 and 6: Primer 3 for wild and mutant K12; lane 7: 10000 bp DNA ladder; lane 8 and 9: Primer 4 for wild and mutant K12.

After isolating the selected mutant, we investigated the impact of mutagenesis on the genome of the coding region of HAS from the upstream area of the hasA promoter to hasC. This allowed us to compare the wild type (designated as K1) with the selected mutant (mutant NU-1-36 designated as K12). The results for both the wild-type and the mutant strains have been registered on the NCBI website (accession numbers PQ310577 for K1 and PQ310578 for K12). Successful amplification of the target regions was confirmed by agarose gel electrophoresis before sequencing (Figure 2). The gel image revealed distinct bands corresponding to the expected sizes for each primer set: Approximately 1 kb for primers 1 and 2, and approximately 1.2 kb for primers 3 and 4. Specifically, bands of approximately 972, 1002, 1217, and 1229 bp were observed for PCR products from primer sets 1, 2, 3, and 4, respectively.

Sequencing of the HA synthesis genes A, B, and C (main enzymes for HA production) revealed a single nucleotide polymorphism between the wild type (K1) and the selected mutant (K12). At the 889th nucleotide of HasA, a "G" in the wild-type strain is replaced with a "T" in the mutant strain. This single-nucleotide polymorphism results in an amino acid change at position 248 of the HasA protein sequence, where the histidine (H) in K1 is substituted with glutamine (Q) in K12.

### 4.4. Molecular Weight Analysis

**Figure 3.**
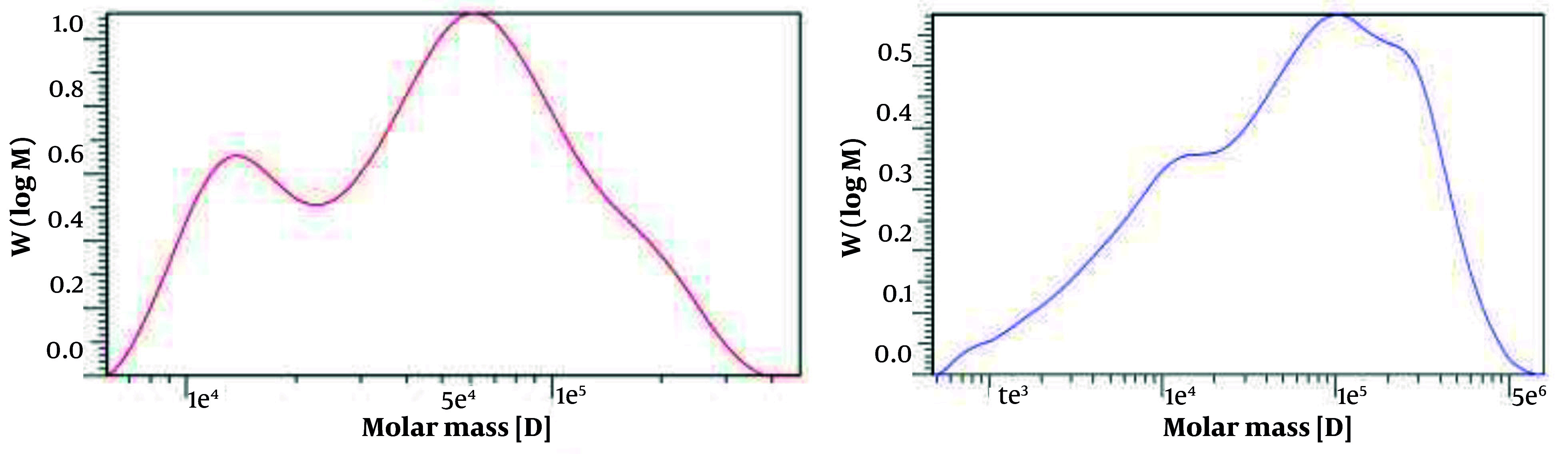
Gel permeation chromatograph of K1 (Left) with average molecular weight (Mw) 6.7 × 10^4^ Da and K12 (Right) with average Mw 1.2 × 10^5^ Da derived hyaluronic acid (HA) Mw characteristics

As shown in Figure 3 for the HA obtained from K1 (left) and K12 (right), the average Mw of the sample was determined to be 6.6960 × 10^4^ Da and 1.2373 × 10^5^ Da, respectively, indicating the average size of the HA molecules. The number-average Mw (Mn) value, which represents the statistical average of the Mws of individual polymer chains, was 3.1275 × 10^4^ Da and 1.2826 × 10^4^ Da, respectively.

### 4.5. Comparison of the Impact of Different Pre-treatment Procedures

**Figure 4.**
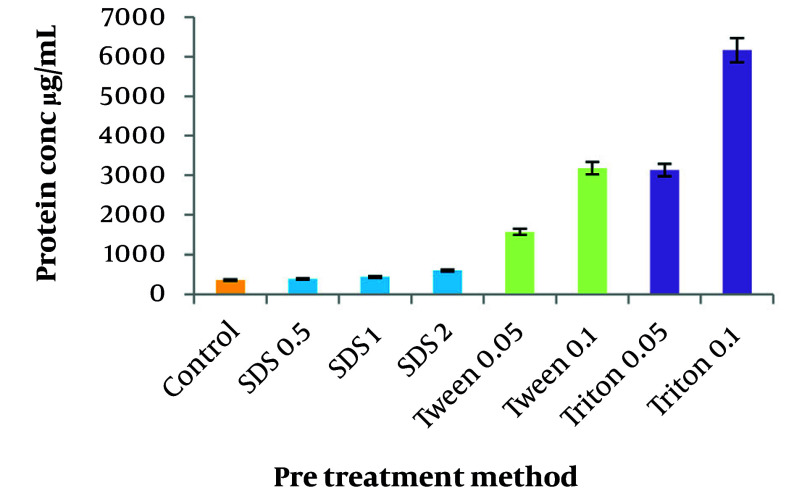
Effects of different pre-treatment methods on protein impurity

The efficiency of pre-treatment methods using three different detergents was compared in terms of protein removal. As shown in Figure 4, all detergents prevented protein denaturation and aggregation by preserving the protein structure and dissolving membrane proteins, resulting in an increase in protein content. Among the detergents, SDS exhibited the lowest increase in protein impurity at 23.6%, followed by Tween 20 and Triton X100, which contributed to a higher level of protein impurities compared with the no pre-treatment method.

### 4.6. Protein Removal and Hyaluronic Acid Recovery Through pH and Temperature Adjustment

**Figure 5.**
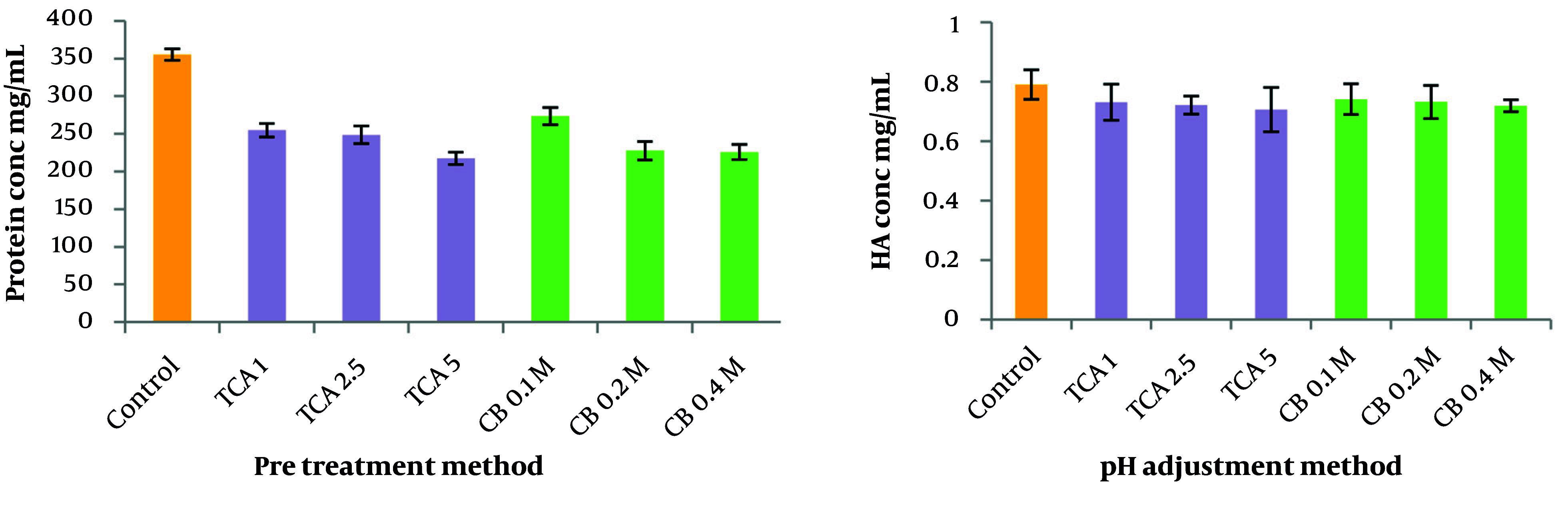
Effects of pH adjustment with TCA or citrate buffer on protein removal (Left) and hyaluronic acid (HA) recovery (Right)

The study compared the effectiveness of TCA and citrate buffer as the first purification step in terms of HA recovery and protein removal. The results demonstrated the superiority of citrate buffer over TCA in terms of minimizing HA loss. Among the three concentrations of citrate buffer tested (0.1 M, 0.2 M, and 0.4 M), it was observed that 0.1 M citrate buffer reduced HA recovery by 5.8%, whereas 0.2 M reduced HA recovery by 7%. Despite the slightly higher HA loss with 0.2 M citrate buffer, this concentration was selected as the optimal pre-treatment method because of the superior protein removal efficiency of 0.2 M citrate buffer, which achieved 35% protein removal compared to 23% for the 0.1 M concentration (Figure 5). 

In terms of examining the effects of temperature, the results indicated that when the acidified culture was maintained at 4°C, the system demonstrated a 33.7% greater reduction in protein impurities and a 4% increase in HA recovery compared with the 60°C condition. These findings underscore the superior efficacy of low-temperature conditions for enhancing protein purification and maximizing HA yield.

### 4.7. Protein Removal and Hyaluronic Acid Recovery Throughout UF

**Figure 6.**
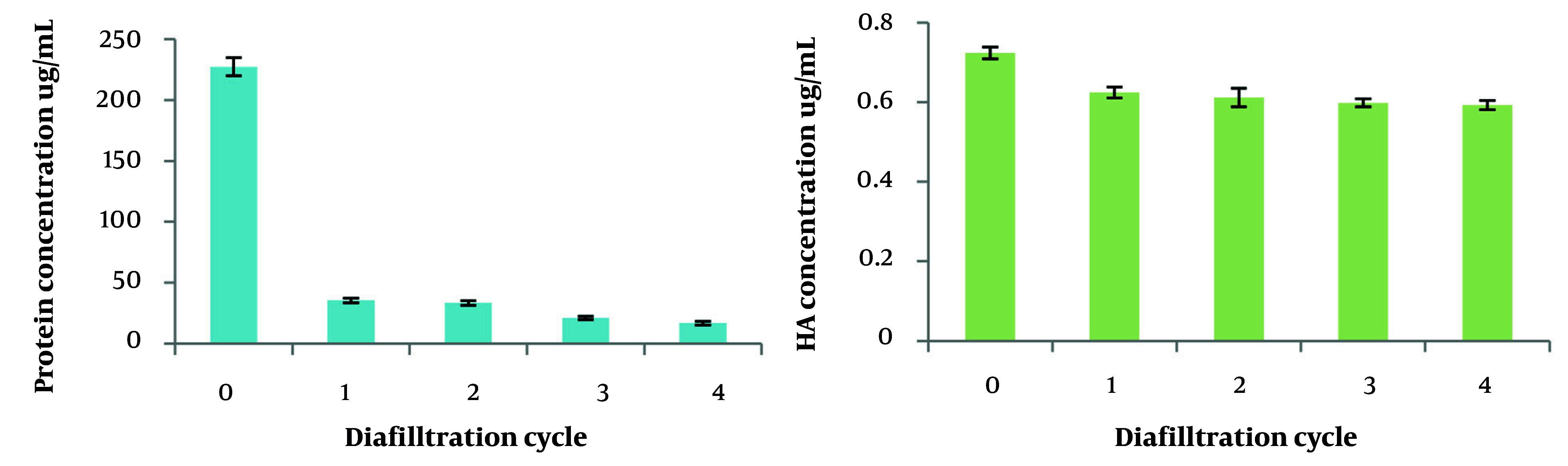
UF efficacy on protein concentration (Left) and hyaluronic acid (HA) concentration (Right) throughout 4 diavolume changes

In the UF experiments conducted over four diavolume changes, significant reductions in protein impurities were observed, with removal efficiencies of 84.4%, 85.3%, 90.7%, and 92.6% for the first retentate through the fourth, respectively. However, it is important to note that there was a slight concomitant loss of HA during this process, with reductions measured at 13.7%, 15.5%, 17.3%, and 18.1% across the four cycles. Figure 6 illustrates these results, highlighting the balance between protein impurity removal and HA retention throughout the UF steps.

### 4.8. Phenol Acetate Treatment

Following UF, a phenol acetate treatment was employed as a final polishing step to further reduce protein impurities in the collected retentate solution. During this process, HA accumulates in the aqueous phase, which is then collected and freeze-dried to obtain HA powder suitable for Lowry protein quantification. This treatment significantly reduced the protein content from 2.8% to 0.27%, as determined by the Lowry test. The resulting HA product met the EP standards for pharmaceutical-grade purity, with protein impurities well below the 0.3% threshold.

### 4.9. Characterization of Purified Hyaluronic Acid

#### 4.9.1. Hyaluronic Acid Molecule Confirmation

**Figure 7.**
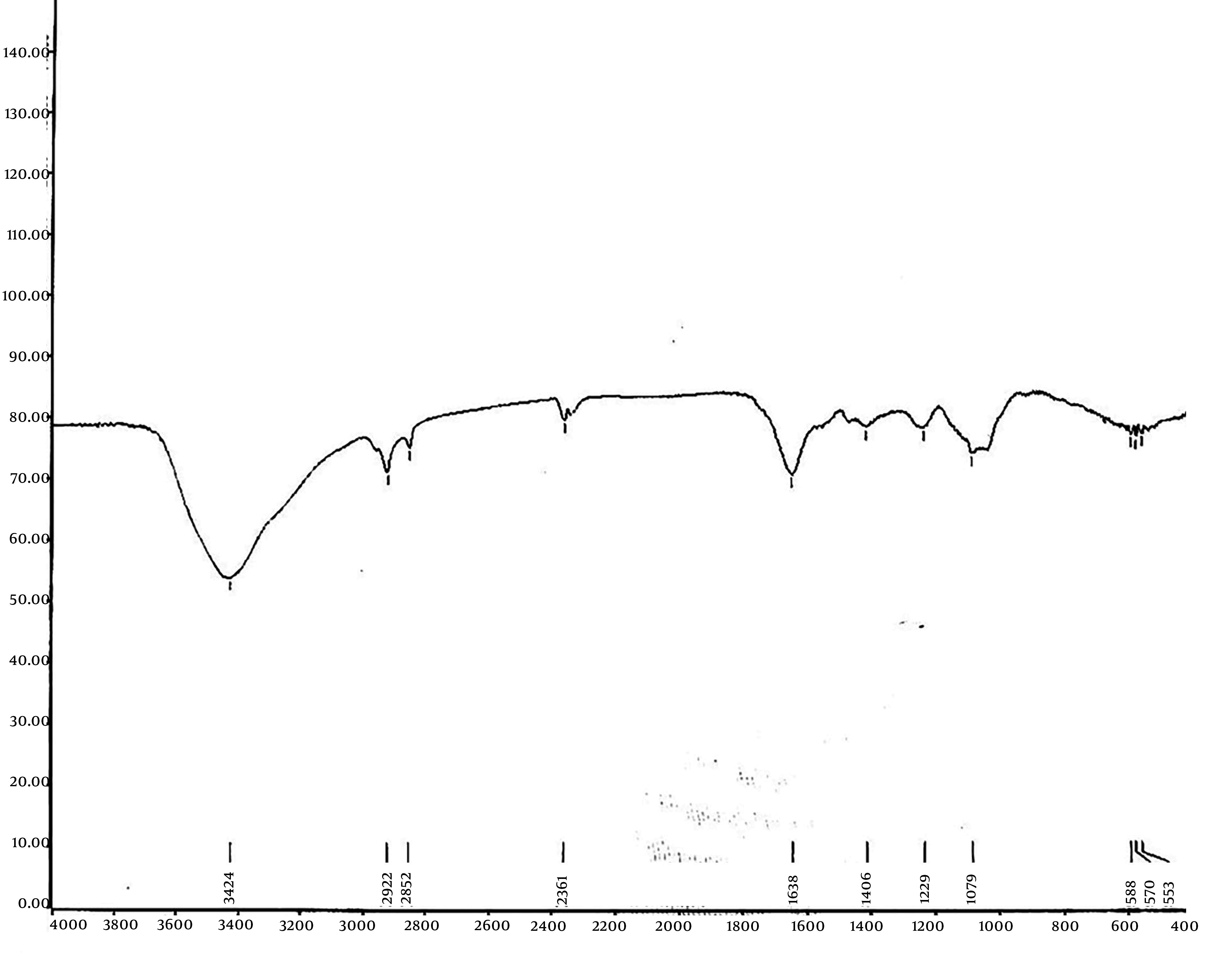
Fourier transform infrared (FTIR) characterization of hyaluronic acid (HA) produced by mutant *Streptococcus zooepidemicus* K12

To characterize the purified HA, an FTIR spectrophotometer was used, scanning within the range of 400 to 3000 cm^-1^. As illustrated in Figure 7, the FTIR spectrum of HA produced by *S. zooepidemicus* demonstrates strong structural alignment with reference standards and literature reports (23, 24). Our spectrum shows a prominent peak at 3424 cm^-1^, corresponding to OH stretching and N-H vibrations in the N-acetyl side chain, which closely matches the 3435 cm^-1^ peak reported for OH/NH stretching in HA and aligns with the 3348 cm^-1^ band observed in conventional HA. Minor shifts may arise from differences in hydrogen bonding or sample hydration. The bands at 2922 cm^-1^ and 2652 cm^-1^ in our study, which are attributed to symmetric methyl C-H stretching in glucuronic acid, correlate well with the 2919 cm^-1^ (CH/CH_2_) and 2926 - 2927 cm^-1^ (glucuronic acid) peaks. These variations likely reflect differences in crystallinity or intermolecular interactions. Peaks at 1638 cm^-1^ (C=O carboxyl) and 1406 cm^-1^ (primary aromatic amine CN stretching) in our analysis align with literature values for amide I (1632 cm^-1^, 1637 cm^-1^) and symmetric C-O stretching (1416 cm^-1^, 1421 cm^-1^), confirming the preservation of HA’s polysaccharide backbone and amide functionalities. Additionally, the major alcohol C-O stretch observed at 1079 cm^-1^ in our study falls between the 1084 cm^-1^ (carbohydrate C-O) and 1023 cm^-1^ (alcohol C-O) reported in literature, potentially due to differences in Mw. These spectral features are consistent with the established standard FTIR graph for HA, confirming that our monograph accurately captures the molecular structure and identity of HA.

#### 4.9.2. Chromatography of Simultaneous Protein and Hyaluronic Acid Peak Analysis

**Figure 8.**
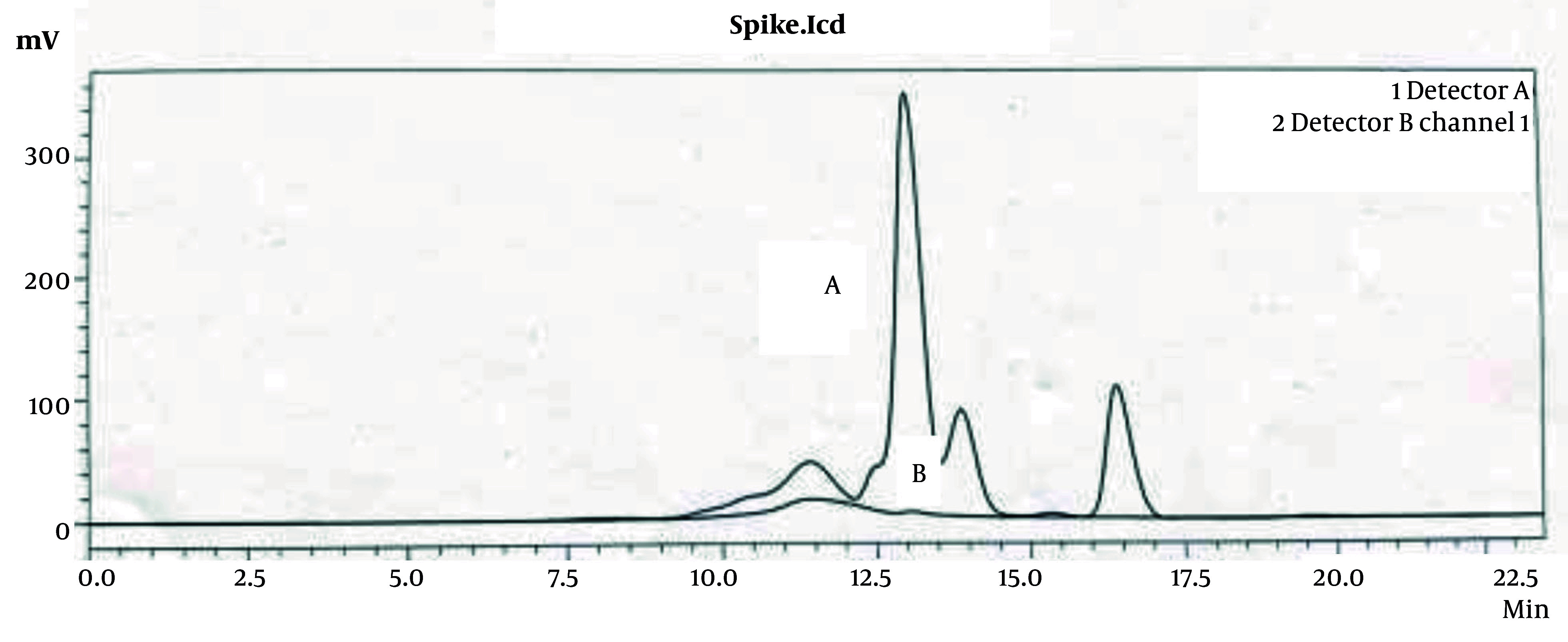
Size exclusion chromatograms of the injected hyaluronic acid (HA) sample in hydrogel 2000 column: The plots A and B show RI detection signal and UV absorption at 280 nm, respectively.

Analysis using size exclusion chromatography with a hydrogel 2000 column revealed a significant reduction in protein impurities in relation to the HA peak. As shown in Figure 8, the chromatograms indicate an almost complete omission of the protein impurity peak, as evidenced by the very low absorbance at 280 nm. This demonstrates the effectiveness of the downstream method in selectively isolating HA while minimizing protein contamination, thereby enhancing the purity of the final product.

#### 4.9.3. Measurement of DNA Impurities

The absorbance at 260 nm for the 100 mg freeze-dried HA sample in a 9 g/L sodium chloride solution was measured at 0.584 based on three repetitions. This value is on the border of the EP standard, indicating that it should be less than 0.5.

#### 4.9.4. Phenol Residue Quantification

According to the EP method 2.5.15, the phenol concentration in the purified HA sample was measured at 0.086% w/w.

#### 4.9.5. Loss-on-Drying

The LOD analysis revealed that the sample lost 4.09% of its initial weight during the drying process, indicating the percentage of volatile substances or moisture present in the original sample, which passed the 20% limitation based on EP.

## 5. Discussion

Random mutagenesis is an effective approach for strain improvement in various microorganisms, including *S. zooepidemicus*. According to Kim et al. (17), a high Mw HA -producing mutant, designated *S. equi* KFCC 10830, was developed from *S. equi* ATCC 6580 through serial selection following NTG treatment. The selected mutant exhibited non-hemolytic and hyaluronidase-negative characteristics, along with kanamycin resistance and high viscosity. Zhang and Feng (6) developed a mutant strain of *S.* *equi*, designated JC-63, capable of producing high Mw HA. This strain was derived from *S. equi* JC-O via a compound mutation breeding process using mutagenic agents such as 5-bromouracil, UV radiation, and NTG. After 25 generations of inoculation, *S. equi* JC-63 exhibited stable inheritance and non-hemolytic characteristics.

Our mutant strain of *S.* *equi* subsp. *S. zooepidemicus* K12 exhibited an 85.7% increase in HA production, rising from 0.42 g/L to 0.78 g/L through combined UV and chemical mutagenesis. Additionally, the Mw of HA increased from 6.7 × 10^4^ Da to approximately 1.2 × 10^5^ Da, enhancing its biological and industrial applicability. At this Mw, HA exhibits enhanced skin penetration and hydration capabilities, making it particularly effective in cosmetic products aimed at deep moisturizing and anti-aging benefits. Additionally, HA of this size supports improved viscoelasticity and tissue compatibility, which are advantageous for wound healing and regenerative medicine applications (25). This relative improvement aligns with the findings of Jafari et al. (7), who reported HA yield increases between 45.5% (from 1.24 to 1.80 g/L) and 130% (from 1.24 to 2.86 g/L). Similarly, Yao et al. (26) employed atmospheric and room temperature plasma (ARTP) mutagenesis with high-throughput screening, achieving a 42.9% increase in HA yield, reaching 0.813 g/L in shaking flask culture and scaling up to 4.56 g/L in a 5-L fermenter. Although these mutants achieved higher absolute HA titers, our study highlights a significant relative yield enhancement from a lower starting point.

Sequencing of the HasA gene of *S. zooepidemicus* revealed a single nucleotide substitution between the wild-type and mutant strains. At the 889th nucleotide of HasA, a "G" in the wild-type strain is replaced with a "T" in the mutant strain. This single nucleotide polymorphism results in an amino acid change at position 248 of the HasA protein sequence, where a histidine (H) in the wild-type is substituted with a glutamine (Q) in the mutant. This amino acid substitution could have substantial implications for protein structure and function. Previous studies have shown that modifications in hyaluronan synthase can affect the Mw and production of HA. For instance, a study by Tlusta et al. (27) demonstrated that specific mutations in the has operon promoter (e.g., AG or GT at positions -49/-50) directly increase HA yields in *S.* *equi* subsp. *S. zooepidemicus*, with strains SEZPhasAG and SEZPhas2G producing 116% and 105% HA, respectively. This demonstrates how modifications in hyaluronan synthase affect the Mw and production of HA. Although the has operon is the key locus responsible for HA production, whole-genome sequencing is still recommended for future studies to comprehensively rule out any off-target mutations that may influence bacterial virulence or growth.

Following the development of an optimized strain, a key focus is purification, which is an essential step in HA production. In some manuscripts, researchers employ a pre-treatment step using SDS before bacterial separation with the aim of facilitating the separation of the HA capsule from the bacterial cell wall. However, according to our findings, using SDS and even non-anionic detergents such as Triton X100 and Tween 20 introduces significant challenges to the purification process. The use of detergents often leads to solubilizing protein membranes and bacterial cell lysis, resulting in the release of intracellular proteins and DNA into the medium, which inadvertently increases the overall impurity profile of the extract. Unlike detergent-based methods, warming the broth to 70°C for 1 hour, a technique similar to that used by Jagadeeswara Reddy and Karunakaran (28) to reduce viscosity and facilitate HA liberation, was chosen as an alternative pre-treatment. Wang et al. demonstrated that heating pretreatment outperformed acidification and dilution by achieving the highest HA recovery (92.46%) while reducing chemical contaminants, improving solution transfer efficiency, and lowering costs (29). Therefore, heat-based pretreatment is preferable over SDS or acid-based methods, which tend to increase protein impurities and reduce HA yield.

In some studies, acids such as TCA or citrate buffer were used in the initial purification step. For example, studies by Cavalcanti and Santana (11) and Cimini et al. (30) highlighted the role of pH and sodium chloride in recovering high Mw bio-HA through precipitation methods. Consistent with these findings, our results (Figure 5) showed that 5% TCA and 0.2 M citrate buffer reduced protein impurities by 38% and 35%, respectively, likely due to induced protein aggregation. However, citrate buffer outperformed TCA in HA recovery, achieving 93% versus 89%. Therefore, citrate buffer was selected as the preferred purification agent in this study.

Ultrafiltration is a widely used method in biotechnology for purifying HA due to its efficiency, mild operating conditions, and environmental benefits compared to solvent precipitation. Traditionally, UF serves as a final polishing step in HA purification, often combined with other methods to remove low Mw and insoluble impurities (31-33). In this study, we applied UF at the initial purification stage, with the aim of reducing or eliminating the need for alcohol precipitation and streamlining the process. The 50 kDa membrane MWCO was chosen based on size-exclusion chromatography results indicating an average HA Mw of about 120 kDa, allowing removal of low Mw HA below 50 kDa that lack the higher value associated with the retained high and medium Mw HA fractions important for pharmaceutical and cosmetic applications, while retaining medium and high Mw HA. Ultrafiltration was performed until reaching a VCR of 4, based on Zhou et al.'s findings that this level optimally balances protein impurity removal and HA recovery (19). Our results demonstrate that UF efficiently removes protein impurities, achieving 84.4% removal in the first diafiltration cycle and up to 92.6% removal after four cycles, confirming its effectiveness as an initial purification step.

The use of phenol acetate precipitation as a polishing step for protein impurity removal in polysaccharide purification has shown promising results in various bioprocessing applications (34) and represents a novel approach to HA purification. This method exploits the differential solubility of proteins and polysaccharides in phenol-containing solutions through the following mechanisms:

1. Protein denaturation: Phenol disrupts the hydrogen bonds and hydrophobic interactions that maintain protein structure, causing proteins to unfold.

2. Phase separation: The addition of acetate buffer creates a biphasic system. Denatured proteins tend to partition into the organic phenol phase, while polysaccharides remain in the aqueous phase.

3. Protein precipitation: The denatured proteins in the phenol phase can be precipitated, further separating them from other biomolecules.

While phenol acetate precipitation has been established for other polysaccharides, its successful implementation for HA purification to pharmaceutical-grade (below 0.3% protein impurity) opens new avenues for more efficient and cost-effective production of high-purity HA. The phenol concentration in the purified HA API form sample prepared in this study was measured at 0.086% w/w, which is within the allowable phenol limit. According to the EP, phenol can be used as a preservative in injectable pharmaceutical finished products up to a concentration of 2.5 g/L (0.25%, equivalent to 2500 ppm) (21). It is worth noting that proper disposal of phenol is important to mitigate environmental concerns and ensure the sustainable application of this method.

The HA production efficiency depends significantly on both microbial strain capabilities and downstream purification methods and varies widely, up to 7 g/L in highly engineered and supplemented systems (7). In this study, we applied an integrated approach combining strain optimization with a tailored purification process to enhance both HA yield and purity. Our mutant *S. equi* subsp. *S. zooepidemicus* K12 strain produced 0.78 g/L of HA with a Mw of 1.2 × 10^5^ Da, and the purified HA met pharmaceutical-grade purity standards.

Compared to previous works, such as Oueslati et al., who worked on *S. equi* subsp. *S. zooepidemicus* and reported a yield of 0.79 g/L with HA of Mw approximately 1.5 × 10^3^ kDa and purity around 90%, their purification method was based on diafiltration (35). Sousa et al. conducted their study using *S. zooepidemicus*, achieving a yield of 0.78 g/L and producing HA with a Mw around 10 × 10^3^ kDa, with pharmaceutical-grade purity. Their purification method involved ethanol precipitation followed by size exclusion chromatography (36).

Our study demonstrates a balanced improvement in both productivity and product quality compared to these benchmarks. Unlike conventional methods that add large volumes of ethanol early (around three times the culture volume), our method first removes protein impurities via pH and temperature adjustments followed by ultrafiltration, reducing volume to less than one-quarter. Subsequent phenol acetate treatment halves the volume before ethanol is added only at the final step. This sequence reduces organic solvent consumption by over 25-fold, enhancing cost-effectiveness and environmental sustainability.

Our simplified purification protocol achieves a final HA recovery rate of 72%, outperforming previous reports like Rangaswamy and Jain (65%) and Jagadeeswara Reddy and Karunakaran (62.5%), while efficiently removing impurities (12, 28). However, considering that the DNA impurity level marginally exceeds the EP limits, and to further improve protein purification, optimizing the final organic solvent precipitation step is recommended in future studies. The literature (11, 15) indicates that factors such as the ratio and type of organic solvent, as well as the duration of incubation at low temperatures, can significantly influence the solubility of DNA and proteins and thus affect the efficiency of purification.

### 5.1. Conclusions

A *S. equi* mutant K12 was generated through random mutagenesis, which lacks hemolytic and hyaluronidase activities, significantly improving HA production yields by removing this catabolic pathway. This study demonstrated the effectiveness of targeting virulence genes via random mutagenesis as a strategy for strain improvement in biotechnology applications. Following strain optimization, an efficient downstream process was implemented to minimize organic solvent consumption. This process involved pH and temperature adjustments, followed by ultrafiltration and phenol acetate treatment. The simplicity of the proposed method, combined with its effective recovery rate, demonstrates its potential as a viable alternative to more complicated and solvent-intensive purification strategies.

## Data Availability

The data presented in this study are uploaded during submission as a supplementary file and are openly available for readers upon request.
